# Tobacco straw biochar enhances pak choi growth and quality: Lead/cadmium immobilization and soil fertility improvement

**DOI:** 10.1016/j.isci.2026.116274

**Published:** 2026-06-11

**Authors:** Xue Tian, Faguang Li, Jianghu Long, Yang Luo, Mengyang Wang, Jie Cheng, Luqing Yang, Xiongyan Yang, Zixia Song

**Affiliations:** 1 School of Geography and Resources, Guizhou Education University, Guiyang 550018, China

**Keywords:** Environmental science, Environmental technology

## Abstract

To address heavy metal contamination in farmland and promote tobacco straw waste utilization, this pot experiment compared three application rates (1%, 3%, 5%) of tobacco straw biochar on pak choi growth, quality, nutrient and heavy metal uptake, and soil properties and bacterial communities. The results showed that, compared to the control, the addition of biochar increased shoot fresh weight by 15.56–18.97%, and contents of soluble sugar, soluble protein, and vitamin C by up to 109.82%, 62.03%, and 153.36%, respectively. Biochar addition reduced soil bulk density, increased pH, organic matter, alkali-hydrolyzable nitrogen, and available potassium, and reduced Cd and Pb contents in pak choi shoots by 30.76% and 38.98%, respectively. Furthermore, biochar addition significantly increased the Shannon index of the soil bacterial community and the relative abundances of Gemmatimonadota, Myxococcota, and norank_Saprospiraceae. Based on a comprehensive evaluation, the 3% application rate was the most effective.

## Highlights

• Tobacco straw biochar promoted the growth of pak choi in Pb-Cd contaminated soil
• Tobacco straw biochar increased sugar, protein, vitamin C, and reduced nitrate in pak choi
• At 3–5% application rates, tobacco straw biochar reduced heavy metal uptake in pak choi
• Tobacco straw biochar improved soil physicochemical properties and bacterial diversity

## Graphical abstract

**Figure:**
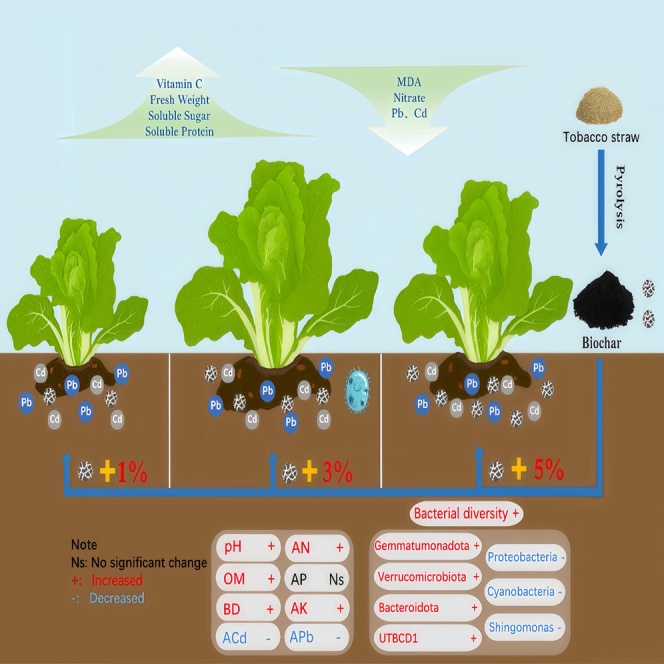

## Introduction

Heavy metals enter and accumulate in soil through pathways such as mining, smelting, sludge discharge, and fertilizer application, deteriorating the soil environment. Survey results indicate that approximately one-sixth of China’s agricultural land is contaminated by heavy metals such as cadmium (Cd) and lead (Pb).^1^ Heavy metals in soil interfere with the normal metabolic functions of crops by inducing oxidative stress, damaging enzyme systems, antagonizing nutrient elements, and impairing cellular structures, inhibiting growth and lowering quality.^2^ These metals eventually threaten human health through transfer along the food chain. Therefore, the implementation of effective remediation measures to restrict the translocation of heavy metals from soil to crops is imperative for ensuring the safe use of contaminated farmland and raising sustainable agricultural development.

Biochar is a highly aromatic and refractory solid material produced through the pyrolysis and carbonization of biomass under partial or complete oxygen limitation. It has gained substantial attention in recent years for remediating heavy metal-contaminated soil because of its well-developed pore structure, large surface area, abundance of functional groups, and high nutrient content. A two-year field experiment conducted in a tea garden with soil pH below 4.5 and contaminated by Pb and Cd showed that biochar addition inhibited the transfer of heavy metals in the soil-plant system, resulting in a significant reduction of Pb and Cd content in tea leaves by 61.28% and 28.57%, respectively.^3^ A meta-analysis on the immobilization of heavy metals in soil by biochar found that biochar application alone could reduce the availability of soil heavy metals Cd and Pb by 24.0% and 31.3%, respectively.^4^ However, the effectiveness of biochar is not uniform and is primarily influenced by several factors: (1) Biochar application rate. Excessive application not only raises remediation costs but can also enlarge soil pores excessively, reduce water retention, and disrupt nutrient balance. In contrast, insufficient application limits its ability to enhance soil structure and immobilize heavy metals.^5^^,^^6^ (2) Feedstock used for biochar production. Due to differences in raw material composition, biochars derived from distinct feedstocks often display varying adsorption capacities for heavy metals even at identical application rates.^7^ (3) Type and contamination level of heavy metals. Due to differences in ionic radius, charge density, electronegativity, hydrolysis constant, and complexation ability, heavy metal ions exhibit distinct interaction modes and binding strengths with functional groups on the biochar surface.^8^

China is the world’s largest tobacco producer, with a tobacco planting area of 10.4356 million hectares and an output of approximately 2.19 million metric tons in 2022. Tobacco straw is the main waste generated during tobacco production, accounting for about 48% of the total tobacco yield.^9^ Current disposal methods for tobacco straw include the following: (1) Incineration. This approach releases large amounts of smoke and harmful gases, polluting the air and endangering respiratory health.^10^ (2) Random stacking. This practice occupies land resources and raises decomposition, fostering pests and diseases. (3) Manufacture of synthetic panels. Due to its high-water absorbency and low toughness, tobacco straw is prone to cracking, deformation, and mold growth. Tobacco straw contains significant amounts of cellulose, organic carbon, and lignin, along with abundant mineral elements. As the primary component, cellulose facilitates the development of a porous structure during pyrolysis, thereby enhancing the biochar’s specific surface area and adsorption capacity. A high organic carbon content promotes the formation of a stable carbon skeleton after carbonization, favoring soil carbon sequestration and durable soil improvement. Lignin, under relatively high pyrolysis temperatures, contributes to the formation of highly aromatized carbon structures, which increases the biochar’s chemical stability and its complexation ability with contaminants. Furthermore, abundant mineral elements such as potassium (K), calcium, and magnesium are largely preserved in the biochar as oxides or carbonates post-carbonization. These components can slowly release nutrients into the soil, while also regulating soil pH and affecting heavy metal speciation via processes such as ion exchange and precipitation. Previous studies have demonstrated that applying tobacco straw biochar can improve soil fertility and enhance soil microbial community structure.^11^^,^^12^ However, studies addressing the immobilization effectiveness of tobacco straw biochar in heavy metal-contaminated soil remain relatively limited.

Pak choi (*Brassica rapa* subsp. *chinensis*), an important leafy vegetable of the Brassicaceae family and Brassica genus, is rich in nutrients such as vitamin C, calcium, iron, and K. It is among the most widely consumed leafy vegetables in China. In addition, owing to its short growth cycle, high biomass, and high sensitivity to heavy metal stress, pak choi is often used as a model crop in soil pollution toxicology studies.^13^^,^^14^ This study conducts a pot experiment cultivating pak choi in Pb-Cd co-contaminated soil to evaluate the effects of tobacco straw biochar amendment on its growth and quality characteristics. Simultaneously, by analyzing changes in physiological indicators of pak choi, nutrient indicators, soil physicochemical properties, and soil bacterial community structure, the intrinsic mechanisms of the biochar’s ameliorative effects are clarified. This research aims to provide a scientific foundation for the resource utilization of tobacco straw, the safe production of pak choi, and the remediation of composite heavy metal contamination in soils.

## Results

### Characterization results of tobacco straw biochar

**Figure 1:**
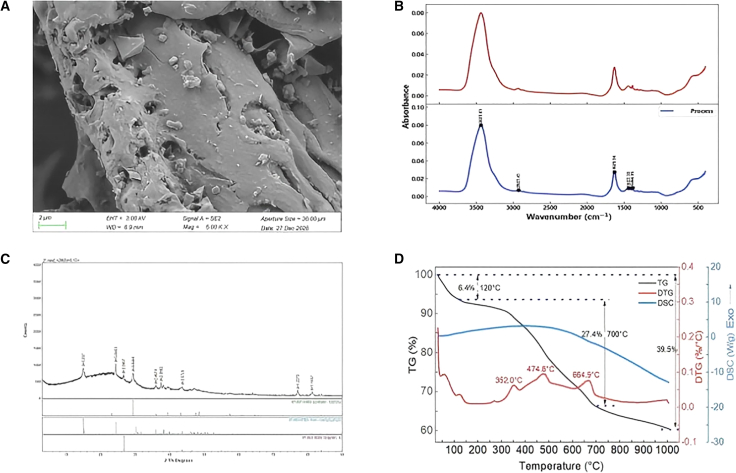
Characterization results of tobacco straw biochar (A) SEM image (scale bars, 2 μm). (B) FTIR spectrum. (C) XRD pattern. (D) TG-DSC curves. Data are mean ± SD.

The SEM results showed that the tobacco straw biochar surface exhibited a rough, lamellar structure with distinct particle protrusions and depressions. Numerous pores of varying sizes were dispersed throughout, and localized areas contained elongated fissures (Figure 1A). Figure 1B presents the FTIR spectrum of the tobacco straw biochar. The broad and strong absorption peak at 3437.01 cm^−1^ is attributed to the O-H stretching vibration. The peak at 2927.41 cm^−1^ is assigned to the C-H stretching vibration (specifically -CH_2_). The absorption peak at 1629.56 cm^−1^ corresponds to characteristic functional groups in aromatic compounds. The peaks at 1437.19 cm^−1^ and 1384.16 cm^−1^ are associated with the asymmetric vibration of carbonate groups or the symmetric stretching vibration of ionized carboxyl groups (-COO-).

The XRD pattern of the tobacco straw biochar is shown in Figure 1C. The broadened (002) diffraction peak located at approximately 2θ = 24.26° corresponds to an interlayer spacing of 0.366 nm, indicating loose stacking of carbon layers with low structural ordering. Quantitative analysis using the Rietveld refinement method revealed that the biochar was primarily composed of amorphous carbon (91.3%), with minor mineral phases such as CaCO_3_ (2.4%) and Ca(C_2_O_4_)(H_2_O) (5.0%). Figure 1D displayed the curves of mass loss and heat flow for the tobacco straw biochar as a function of temperature. The total mass loss of the sample during the heating process was 39.5%, and its thermal decomposition behavior could be divided into three stages. Stage I (30°C–120 °C): Within this temperature range, the sample lost approximately 6.4% of its mass, and the mass loss curve was relatively gentle, indicating a low mass loss rate. The mass loss in this stage was primarily attributed to the desorption and volatilization of adsorbed water and residual solvents. A distinct mass loss peak observed in the 100°C–120 °C range suggested the possible removal of crystalline or bound water, or the gradual release of residual low-boiling-point solvents during heating. Stage II (120°C–700 °C): The sample underwent significant thermal decomposition in this interval, with a cumulative mass loss of approximately 27.4%. The derivative thermogravimetry (DTG) curve displayed three pronounced peaks of mass loss rate at 352.0 °C, 474.8 °C, and 664.9 °C. Stage III (700°C–1000 °C): The sample continued to decompose in the high-temperature range, with a mass loss of about 5.7%. No distinct endothermic or exothermic peaks were observed in the DSC curve over the entire tested temperature range.

### The growth performance of pak choi

Table 1 indicates that the application of tobacco straw biochar increases leaf width in pak choi. At application rates of 3% (B2) and 5% (B3), leaf width significantly rises by 25.65% and 25.07%, respectively, compared to the control (CK). As the application rate of tobacco straw biochar increases, leaf length also shows an upward trend; however, a significant difference from the control was only observed at the 5% application rate (*p* < 0.05). The results also indicated that the 1% application rate did not significantly affect the fresh weight of the shoot parts of pak choi. In contrast, at application rates of 3% and 5%, the fresh weight significantly increased by 15.56% and 18.97%, respectively, compared to the control (*p* < 0.05), although no significant difference was observed between the B2 and B3 treatments.

**Table 1:** Growth indicators of pak choi under different treatments

Treatments	Leaf width (cm)	Leaf length (cm)	Shoot Fresh weight (g)
CK	6.90 ± 0.70b	11.00 ± 1.06b	45.76 ± 2.93b
B1	7.33 ± 1.37ab	11.37 ± 1.31ab	39.49 ± 4.58b
B2	8.67 ± 0.47a	12.60 ± 0.26ab	52.88 ± 2.55a
B3	8.63 ± 0.55a	13.67 ± 1.78a	54.44 ± 2.62a

Note: Different lowercase letters within the same column indicate statistically significant differences among treatments (*p* < 0.05). The same as later in discussion.

### Nutritional quality of pak choi

When the application rate of tobacco straw biochar was 1%, the soluble sugar and soluble protein contents in the leaves of pak choi showed no significant differences compared to the control group (Table 2). However, when the application rate increased to 3% and 5%, the soluble sugar content rose significantly by 105.79% and 109.82%, respectively, while the soluble protein content increased significantly by 62.03% and 44.79% (*p* < 0.05) compared to the control. No significant differences were observed between the B2 and B3 treatments. The vitamin C content in pak choi increased with higher biochar application rates, ranging from 68.18% to 153.36%, with significant differences among all treatments. Compared to the control, the application of biochar also significantly reduced the nitrate content in pak choi leaves, with reductions of 12.18% (B1), 55.24% (B2), and 58.77% (B3). In addition, nitrate contents in the B2 and B3 groups were significantly lower than those in the B1 group.

**Table 2:** Nutritional quality of pak choi under different treatments

Treatments	Soluble sugar (mg/g)	Soluble protein (mg/g)	Vitamin C (mg/kg)	Nitrate (mg/kg)
CK	5.70 ± 0.94b	8.06 ± 0.35b	85.87 ± 3.72days	2470.14 ± 117.39a
B1	6.83 ± 0.62b	7.60 ± 0.37b	144.42 ± 7.02c	2169.27 ± 107.90b
B2	11.73 ± 0.54a	13.06 ± 1.54a	197.56 ± 8.69b	1105.64 ± 120.28c
B3	11.96 ± 0.48a	11.67 ± 1.32a	217.56 ± 13.15a	1018.33 ± 81.05c

### Physiological indicators of pak choi

**Figure 2:**
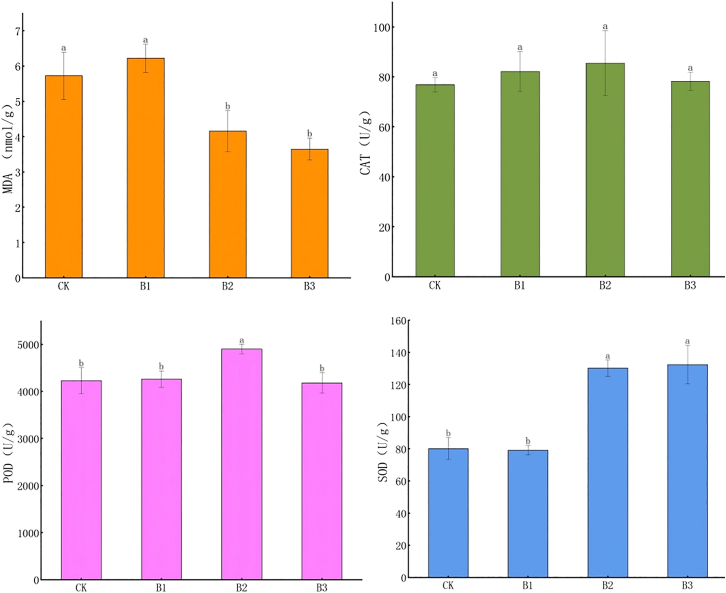
MDA and antioxidant enzyme activities in pak choi Data are mean ± SD. Different letters indicate significant differences at *p* < 0.05 (LSD test).

Figure 2A shows that the malondialdehyde (MDA) content in pak choi leaves exhibited no significant difference between the B2 and B3 treatments but decreased significantly by 27.40% and 36.30%, respectively, compared to the control. The catalase (CAT) activity in pak choi leaves ranged from 76.92 to 85.58 U/g (Figure 2B), with no significant differences among the treatments (*p* > 0.05). After the application of 3% biochar, the peroxidase (POD) activity in pak choi leaves reached its maximum (Figure 2C) and was greater than that in all other treatments. The superoxide dismutase (SOD) activity in pak choi leaves followed the order B3>B2>CK>B1 (Figure 2D). No significant differences were detected between B2 and B3 or between CK and B1, whereas the former two groups showed higher values than the latter two (*p* < 0.05).

### Nutrient and Pb-Cd content in pak choi

**Figure 3:**
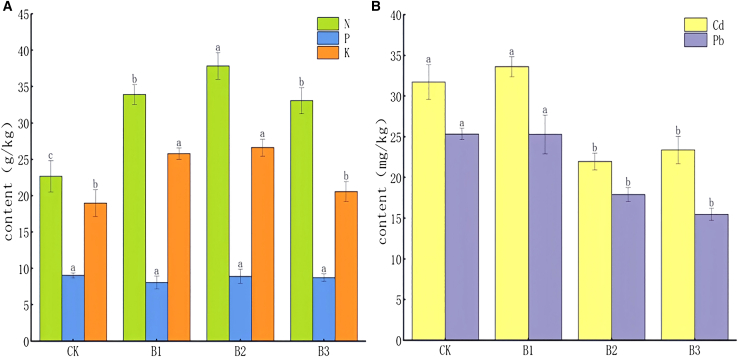
Nutrient and heavy metal contents in pak choi Nutrient contents (A) and heavy metal contents (B) in pak choi shoots. Data are mean ± SD. Different letters indicate significant differences at *p* < 0.05 (LSD test).

The nutrient contents in the shoot parts of pak choi were analyzed, and the results are presented in Figure 3A. The nitrogen (N) content in the shoot parts of the control group was 22.69 g/kg. After the application of tobacco straw biochar, the amount increased significantly to a range of 33.08–37.82 g/kg. Among the treatments, the N content in the B2 group was significantly higher than that in both the B1 and B3 groups. The phosphorus (P) content ranged from 8.07 to 9.06 g/kg, with no significant differences among the treatments (*p* > 0.05). The K content initially increased and then decreased with increasing application rates of tobacco straw biochar. Specifically, the K content in the B1 and B2 treatments increased significantly by 35.79% and 40.21%, respectively, compared to the control (*p* < 0.05). In contrast, the K content in the B3 treatment was significantly lower than that in the B1 and B2 treatments but showed no significant difference compared to the control.

Figure 3B illustrates the heavy metal contents in the shoot parts of pak choi under different treatments. At the 1% application rate of tobacco straw biochar, the Pb and Cd contents showed no significant differences compared to the control (*p* > 0.05). At the 3% application rate, the Pb and Cd contents decreased significantly by 29.44% and 30.76%, respectively, compared to the control. At the 5% application rate, the Pb and Cd contents reduced significantly by 38.98% and 26.28%, respectively (*p* < 0.05). Statistical analysis further indicated no substantial differences in Pb and Cd contents between the B2 and B3 treatments.

### Correlation analysis between growth and quality indicators and physiological, nutrient, and heavy metal indicators in pak choi

**Figure 4:**
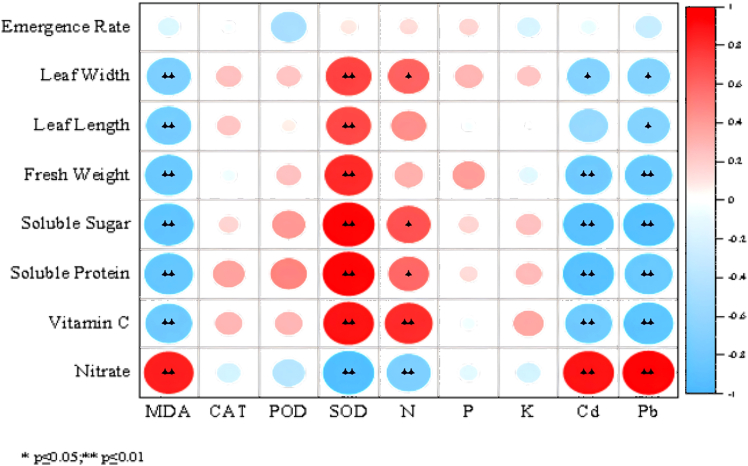
Correlation heatmap of pak choi indicators

Focusing on the growth and quality indicators of pak choi, their correlations with physiological indicators, nutrient indicators, and heavy metal contents were analyzed, and the results are presented in Figure 4. Except for the emergence rate and nitrate content, all other growth and quality indicators of pak choi showed a highly significant negative correlation with the MDA content in the leaves and a highly significant positive correlation with SOD activity (*p* < 0.01). The Cd and Pb contents in the shoot parts of pak choi were highly significantly negatively correlated with fresh weight, soluble sugar, soluble protein, and vitamin C contents (*p* < 0.01), and highly significantly positively correlated with nitrate content (*p* < 0.01). In addition, they showed significant negative correlations with leaf width (*p* < 0.05). The N content in the shoot parts of pak choi was highly significantly positively correlated with vitamin C content and highly significantly negatively correlated with nitrate content (*p* < 0.01). It also demonstrated significant positive correlations with leaf width, soluble sugar, and soluble protein contents (*p* < 0.05).

### Main physicochemical properties and Pb-Cd availability in soil

The effects of different treatments on soil physicochemical properties are shown in Table 3. The application of 3% and 5% tobacco straw biochar significantly reduced soil bulk density (BD) from 1.22 g/cm^3^ in the control to 1.10 g/cm^3^ and 1.03 g/cm^3^, respectively. Only the 5% biochar application significantly altered the soil pH, increasing it by 0.20 units compared to the control. With higher application rates of tobacco straw biochar, the OM content also increased by 9.98%–25.37%. Among these, the difference between the B3 treatment and the control reached a significant level (*p* < 0.05). The AN content initially increased and then decreased with higher biochar application rates, reaching its maximum in the B2 treatment at 1.11 times that of the control, and showing a significant difference compared to the B3 treatment. The AP content ranged from 49.06 to 53.71 mg/kg, with no significant differences among the treatments (*p* > 0.05). After biochar application, the AK content increased by 10.18%–15.24%. Although there were no significant differences among the application rates, the values in the B1 and B2 treatments were significantly higher than those in the control (*p* < 0.05).

**Table 3:** Basic physical and chemical properties of soils under different treatments

Treatments	BD(g/cm^3^)	pH	OM (g/kg)	AN (mg/kg)	AP (mg/kg)	AK (mg/kg)
CK	1.22 ± 0.02a	7.83 ± 0.08bc	21.64 ± 1.05b	77.05 ± 1.22c	52.96 ± 0.38a	223.16 ± 7.67b
B1	1.26 ± 0.05a	7.72 ± 0.16c	23.39 ± 1.16b	82.34 ± 2.55ab	49.06 ± 4.19a	255.00 ± 11.96a
B2	1.10 ± 0.01b	7.96 ± 0.06ab	23.80 ± 0.83b	85.48 ± 3.31a	52.86 ± 1.63a	257.16 ± 24.41a
B3	1.03 ± 0.07b	8.03 ± 0.07a	27.13 ± 1.79a	78.06 ± 2.97bc	53.71 ± 3.48a	245.88 ± 11.7ab

**Figure 5:**
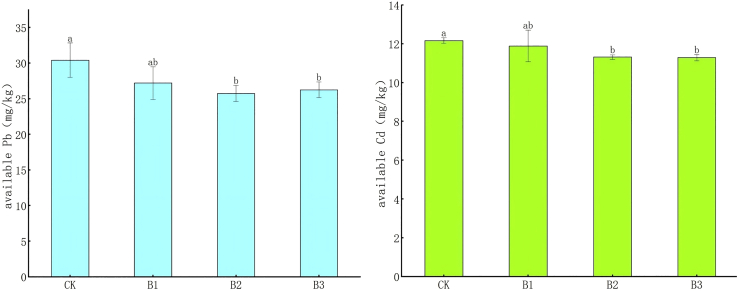
Available Pb and Cd contents in soil Data are mean ± SD. Different letters indicate significant differences at *p* < 0.05 (LSD test).

Figure 5 demonstrates that there were no significant differences in APb and ACd contents among the 1%, 3%, and 5% tobacco straw biochar treatments. However, the extent of change compared to the control varied. Specifically, no difference was observed between the 1% biochar treatment and the control. When the application rate of tobacco straw biochar increased to 3% and 5%, the APb content decreased significantly by 15.32% and 13.68%, respectively, and the ACd content declined substantially by 6.98% and 7.23% compared to the control (*p* < 0.05).

### Correlation analysis between pak choi growth and quality indicators and soil indicators

**Figure 6:**
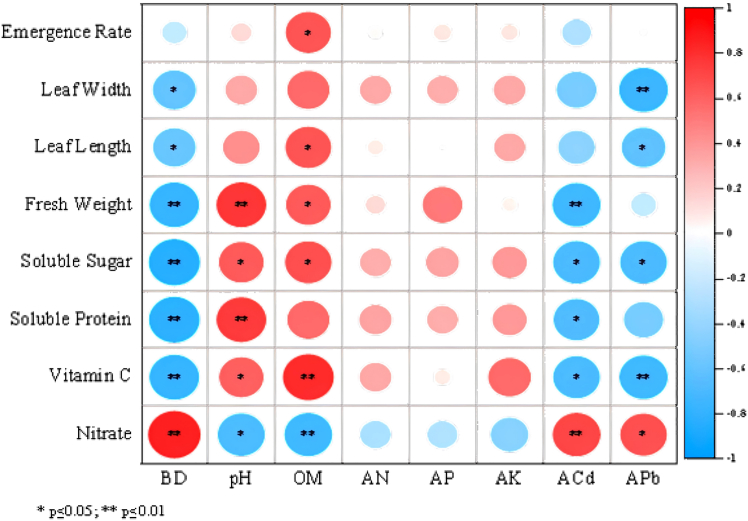
Correlation heatmap between pak choi and soil indicators

Figure 6 shows that soil BD exhibited a highly significant negative correlation with shoot fresh weight, soluble sugar, soluble protein, and vitamin C contents of pak choi (*p* < 0.01), along with a highly significant positive correlation with nitrate content (*p* < 0.01). It was also significantly negatively correlated with leaf length and leaf width (*p* < 0.05). Soil pH showed a highly significant positive correlation with shoot fresh weight and soluble protein content of pak choi (*p* < 0.01), as well as a significant positive correlation with soluble sugar and vitamin C contents (*p* < 0.05). Soil OM content was highly significantly negatively correlated with nitrate content (*p* < 0.01) and highly significantly positively correlated with vitamin C content (*p* < 0.01). It also displayed significant positive correlations with emergence rate, leaf length, leaf width, and soluble sugar content (*p* < 0.05). The ACd content in soil was strongly positively correlated with nitrate content in pak choi (*p* < 0.01) and highly negatively correlated with shoot fresh weight (*p* < 0.01). It also showed significant negative correlations with soluble sugar, soluble protein, and vitamin C contents (*p* < 0.05). The available Pb content in soil was highly negatively correlated with leaf width and vitamin C content (*p* < 0.01), significantly negatively correlated with leaf length and soluble sugar content, and significantly positively correlated with nitrate content in pak choi (*p* < 0.05).

### Soil bacterial community

Table 4 reveals that the Shannon index of the soil bacterial community in all biochar-amended treatments was higher than that of the control, while the Simpson index was lower than that of the control. Among the biochar treatments with different application rates, the Shannon index showed no significant difference. The study also revealed that the Chao index of the soil bacterial community did not vary significantly across the treatments.

**Table 4:** Bacterial community diversity indices of soils under different treatments

Treatments	Shannon	Chao	Simpson
CK	5.87 ± 0.11b	4353.35 ± 271.49a	0.017 ± 0.002a
B1	6.07 ± 0.04a	4457.51 ± 46.63a	0.014 ± 0.002b
B2	6.09 ± 0.06a	4546.81 ± 50.98a	0.012 ± 0.002b
B3	6.12 ± 0.10a	4227.87 ± 201.04a	0.011 ± 0.002b

**Figure 7:**
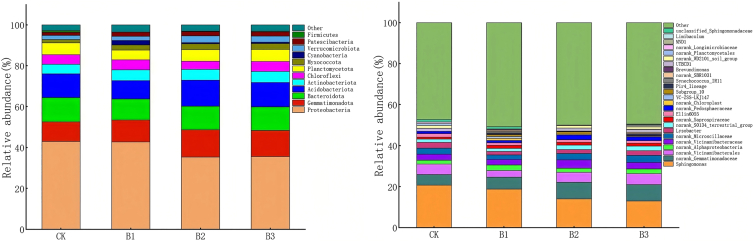
Relative abundances of soil bacteria at phylum (A) and genus (B) levels

The taxonomic composition of the soil bacterial community at the phylum level under different treatments is presented in Figure 7A. Among the 12 major phyla (excluding “other”), Proteobacteria exhibited the highest relative abundance, ranging from 35.31% to 43.06%. The relative abundances in the B2 and B3 treatments were lower than those in the CK and B1 treatments. This was followed by Gemmatimonadota, with relative abundances ranging from 9.49% to 13.42%. The biochar-amended groups were all significantly higher than the control group. Among them, the B2 and B3 treatments were also significantly higher than the B1 treatment group (*p* < 0.05). The relative abundances of Bacteroidota and Acidobacteriota in the soil bacterial community were also relatively high, ranging from 10.24% to 11.94% and 9.01%–12.86%, respectively. However, no significant differences were observed among all treatments. In other phyla, compared with the control, the addition of tobacco straw biochar significantly increased the relative abundance of bacteria belonging to the phylum Myxococcota. The taxonomic composition at the genus level is illustrated in Figure 7B. A total of 25 genera were detected (excluding “other”), with Sphingomonas showing the highest relative abundance, ranging from 13.01% to 20.80%. The B2 and B3 treatments were significantly lower than the CK and B1 treatment groups (*p* < 0.05). Among other genera, the relative abundances of norank_Saprospiraceae in the three biochar-amended groups increased significantly compared to the control, while those of Ellin6055 and Lysobacter decreased significantly. Furthermore, the relative abundances of bacteria belonging to the genera norank_Gemmatimonadaceae and norank_S0134_terrestrial_group also showed a significant increasing trend when the tobacco straw biochar application rates were 3% and 5%.

**Figure 8:**
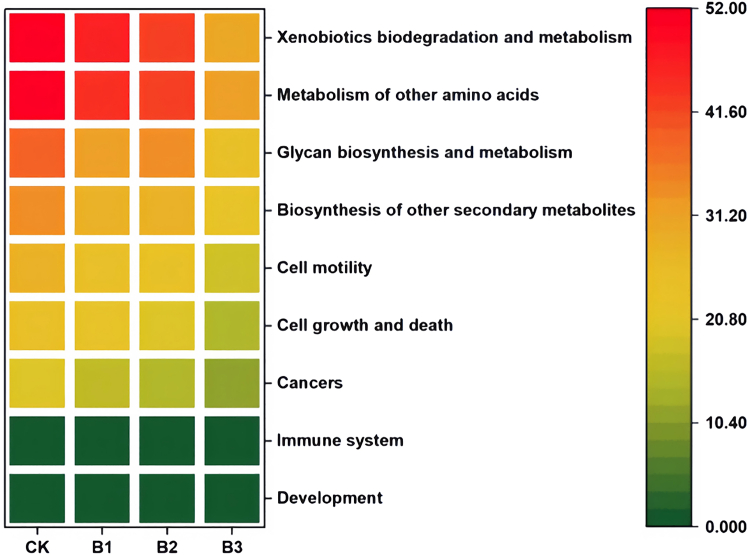
Functional prediction of soil bacterial communities using PICRUSt2

Based on the KEGG database, this study employed PICRUSt2 to predict the functions of non-redundant gene sequences obtained from soils under different treatments, with the results shown in Figure 8. Among the KEGG level 2 pathways, the top nine functions in terms of gene count included xenobiotics biodegradation and metabolism, metabolism of other amino acids, glycan biosynthesis and metabolism, biosynthesis of other secondary metabolites, cell motility, cell growth and death, cancers, immune system, and development, representing the core functions of the bacterial community in the pak choi rhizosphere soil in this study. Compared to the biochar-amended groups, functions such as glycan biosynthesis and metabolism and biosynthesis of other secondary metabolites were stronger in the control group soil bacteria, which may represent a stress response of the bacterial community under Pb and Cd contamination.

### Correlations between bacterial communities and soil environmental factors

**Figure 9:**
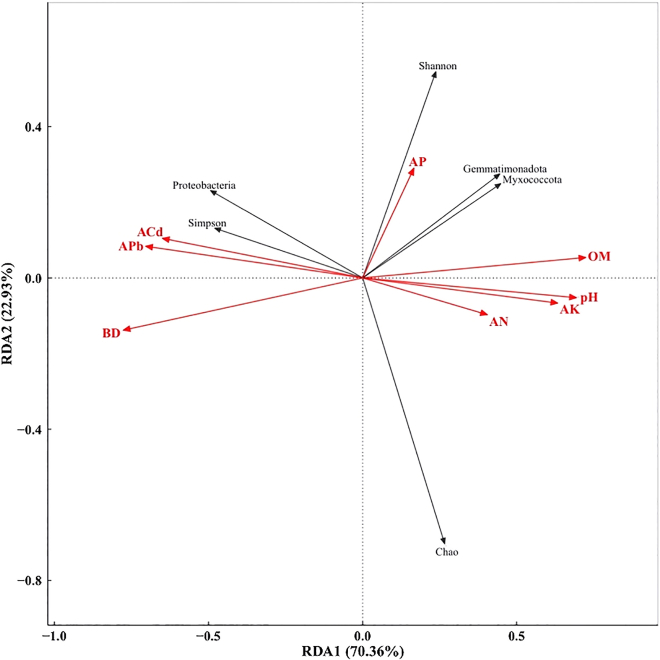
Redundancy analysis (RDA) of soil bacterial community and environmental factors

The redundancy analysis (RDA) results showed that RDA1 and RDA2 explained 70.36% and 22.93% of the species-environment factor relationship variation, respectively, with a cumulative explanation of 93.29%. This indicates that the ordination effectively reflected the coupling relationships between soil bacterial community composition, environmental factors, and diversity indices (Figure 9). Among the soil environmental factors, organic matter (OM), available nitrogen (AN), available potassium (AK), and pH were positively associated with the right side of the RDA1 axis, while available cadmium (ACd), available lead (APb), and BD were oriented toward the left side of the RDA1 axis. Regarding the diversity indices, the Shannon index was positively correlated with AP, AK, pH, and OM. The Chao index was positively correlated with AN, AK, pH, and OM, but negatively correlated with APb and ACd. The Simpson index was positively correlated with APb, ACd, and BD. At the microbial phylum level, Proteobacteria showed a positive correlation with the environmental factors on the left (ACd, APb, BD), whereas Gemmatimonadota and Myxococcota exhibited strong positive correlations with the environmental factors on the right (OM, AN, AK, pH) and with the Shannon index. Judging by the arrow lengths, BD, OM, pH, and the contents of available heavy metals were the main factors driving the soil bacterial community structure.

## Discussion

### Mechanisms of tobacco straw biochar effects on pak choi growth

Analysis of plant growth performance provided direct evidence of the effectiveness of soil amendments. Tran et al.^15^ found that under low copper concentration (50 mg/kg), the application of 1% rice husk biochar increased the aboveground biomass of pak choi by 9.5%, while under high copper concentration (200 mg/kg), an increase of 21.4% was observed (supporting literature). Wang et al.^16^ conducted a pot experiment to ameliorate saline-alkali soil and found that, compared to CK, the sole addition of 2% and 4% corn-straw biochar increased pak choi biomass by 109.92% and 38.43%, respectively. In the present study, following the addition of 3% and 5% tobacco straw biochar, the leaf width of pak choi increased significantly, and the fresh aboveground weight also increased significantly by 15.56% and 18.97% compared to the control. Taken together, it is evident that tobacco straw biochar can exhibit a stable growth-promoting effect at appropriate, low application rates, with its efficacy being comparable to that of some other biochars. However, it must be noted that the soil environment, contamination conditions, and biochar properties involved in different studies vary, so their specific performances should not be simplistically equated and ought to be analyzed within their respective experimental systems. Mechanistically, the reasons for the growth promotion of pak choi by tobacco straw biochar can be attributed to two main factors. First, the addition of biochar improved soil porosity and enhanced soil fertility,^17^ facilitating nutrient uptake by pak choi. Second, as a dicotyledonous plant residue, tobacco straw contains higher lignin content compared to common crop straws such as rice, wheat, and corn.^18^ When converted into biochar, tobacco straw raises the formation of more stable and complex humic molecules, which effectively improve soil structure and interact with soil minerals, releasing essential nutrients that support plant growth and microbial diversity.^19^^,^^20^ In this study, when application rates reached 3% and 5%, soil BD decreased significantly, indicating that tobacco straw biochar improved soil aeration and provided a favorable habitat for bacteria such as Myxococcota and Gemmatimonadota, which play key roles in carbon and N cycling. Their increased relative abundance enhanced nutrient transformation and cycling.^21^^,^^22^ Therefore, the contents of soil OM, AN, and AK were significantly higher than those in the control. Most of these indicators demonstrated significant or highly significant positive correlations with the growth parameters of pak choi.

On the other hand, tobacco straw biochar mitigated the toxic effects of Cd and Pb on pak choi. Once heavy metal elements entered the plants, they penetrated the mitochondria and chloroplasts, directly disrupting the electron transport chain and leading to excessive ROS production.^23^ This excessive ROS damaged biological macromolecules and negatively influenced the antioxidant system of the plants,^24^^,^^25^^,^^26^ inhibiting overall growth. This finding explains why the Cd and Pb contents in pak choi exhibited significant or highly significant negative correlations with indicators such as shoot fresh weight, leaf length, and leaf width. Following the application of 3% and 5% tobacco straw biochar, the uptake of Cd and Pb by pak choi was suppressed, resulting in reduced accumulation in the shoots. Therefore, SOD activity increased significantly by 62.33% and 65.15%, respectively, compared to the control, enhancing the scavenging capacity for ROS. In addition, MDA,^27^^,^^28^ a product of membrane lipid peroxidation under stress conditions, typically demonstrates a negative correlation with plant growth indicators (Figure 3). In this study, the MDA content in the shoots of pak choi under the B2 and B3 treatments decreased significantly compared to the control, indicating that biochar addition improved stress resistance, extended cell membrane stability, and ultimately raised yield enhancement. It is noteworthy that in this study, the functional abundances of glycan biosynthesis and metabolism and biosynthesis of other secondary metabolites in bacteria decreased following biochar addition. This may be attributed to the fact that biochar application immobilized heavy metals, thereby alleviating the stress state of the bacterial community. This shift allowed the community to transition from a high-cost stress metabolism to a more balanced community structure and functional composition.

### Mechanism of tobacco straw biochar’s impact on the quality of pak choi

Their nutritional quality primarily determines the edible value of vegetables. Soluble sugar represents a fundamental indicator of nutritional quality in pak choi, as its content governs sweetness and taste and influences post-harvest preservation capacity.^29^ The results demonstrated that when tobacco straw biochar was applied at 3% and 5%, the soluble sugar content in pak choi shoots increased significantly. This improvement can be attributed to the enhanced water and nutrient retention capacity of the soil following biochar application, which sustained the normal functioning of sugar metabolic pathways.^30^ In addition, the incorporation of tobacco straw biochar increased the relative abundance of Saprospiraceae and Gemmatimonadaceae in the soil. These bacteria play an essential role in decomposing and synthesizing polysaccharide organic substances,^31^ which can further contribute to the elevated soluble sugar content in pak choi leaves.

Soluble protein in vegetables provides readily absorbable essential amino acids for humans, serving as a crucial foundation for tissue building and repair, enzyme, and hormone synthesis, while also contributing a distinct umami flavor to dishes. It is regarded as one of the core indicators of nutritional quality.^32^ In this study, the addition of 3% and 5% tobacco straw biochar significantly enhanced the soluble protein content in pak choi, aligning with the findings of Sofy et al.^33^ This effect can be attributed to the porous structure of tobacco straw biochar, which functions as a slow-release carrier of nutrients, enhances soil N retention,^34^ reduces N loss, and improves the sustained N supply capacity, raising protein synthesis in plants.^35^

Vitamin C serves as a potent antioxidant that scavenges free radicals and strengthens human immunity, and as a vital factor in raising collagen synthesis, enhancing iron absorption, and maintaining skin and vascular health. It is recognized as a key functional component in evaluating the nutritional value of vegetables.^36^^,^^37^ The D-mannose or L-galactose pathway constitutes the primary route for vitamin C biosynthesis, and sugar levels directly regulate its synthesis and metabolism.^38^^,^^39^ Following the application of tobacco straw biochar, the sugar content in pak choi increased. These sugars influenced the biosynthetic pathway of vitamin C by modulating key enzyme activity and regulating the expression of associated genes, which ultimately led to elevated vitamin C content in pak choi.

Nitrate accumulation in vegetables serves as a precursor to nitrosamines, compounds that can be converted into potent carcinogens in the human body.^40^ In this study, as the application rate of tobacco straw biochar increased, the nitrate content in pak choi shoots decreased progressively, reducing associated health risks. This reduction can be attributed to the decreased relative abundance of Sphingomonas, which created a more favorable environment for denitrifying bacteria and raised their proliferation. Therefore, soil nitrate was converted into N or nitrous oxide gas through denitrification and released into the atmosphere. Alternatively, the abundant oxygen-containing functional groups on the surface of biochar can have adsorbed and fixed ammonium nitrogen (NH_4_^+^), limiting its nitrification into nitrate.^41^^,^^42^ In addition, the application of tobacco straw biochar raised shoot biomass accumulation in pak choi, leading to a dilution effect that further contributed to the reduced nitrate content per unit of fresh weight.

### Mechanism of tobacco straw biochar’s impact on lead and cadmium uptake in pak choi

Li et al.^43^ demonstrated that the application of biochar reduced the bioavailability of heavy metals in sewage-irrigated soil, decreasing the concentrations of Cd, Zn, Cu, and other heavy metals in Chinese cabbage, which aligns with the findings of the present study. As the amount of biochar increased, the Pb and Cd contents in the shoots of pak choi significantly decreased. This reduction can be attributed to two primary mechanisms. First, the tobacco straw biochar used in this study possessed a specific surface area of 35.57 m^2^/g, which provided the basis for immobilizing Pb and Cd in the soil through physical adsorption. Simultaneously, results from SEM and FTIR indicated that the biochar surface had abundant pore structures and oxygen-containing functional groups,^44^ which could complex with Cd and Pb in the soil, reducing their bioavailability and thereby inhibiting their accumulation in pak choi (In this study, a significant positive correlation was observed between soil available Pb content and pak choi shoot Pb content,^45^ with a correlation coefficient of 0.671. A highly significant positive correlation was found between soil available Cd content and pak choi shoot Cd content, with a correlation coefficient of 0.717. Second, the tobacco straw biochar in this study had a high pH value of 9.54. When applied at sufficient rates, it could increase the soil pH.^46^ Coupled with the presence of carbonate ions suggested by XRD results, this could promote the formation of hydroxide and carbonate precipitates of Cd and Pb.^47^^,^^48^^,^^49^

Accordingly, the results of this study indicate that varying application rates of tobacco straw biochar differentially influenced the physicochemical properties of the soil, the availability of heavy metals, and the growth and nutritional quality of pak choi. At an application rate of 1%, the effects on most indicators were not significant, likely because the limited amount did not provide sufficient nutrients, porosity, or functional groups to improve soil fertility or enhance heavy metal immobilization. At the 5% application rate, most measured indicators showed no significant differences compared to the 3% rate, indicating that the 3% application can approximate a threshold equilibrium for optimizing soil physicochemical improvement and biological activity regulation. Exceeding this equilibrium point can decrease soil water and nutrient retention capacity and can also result in excessively high C/N ratios and oversaturation of surface functional groups, potentially surpassing the adaptive capacity of soil microorganisms,^50^ negatively affecting the plant growth environment. From an economic perspective, the tobacco straw biochar used in this study demonstrates significant cost advantages and application potential. Its raw material is tobacco agricultural waste, which is widely available and often regarded as an environmental burden. Transforming this waste into biochar achieves a resource cycle of “using waste to treat pollution.” The pyrolysis process is relatively straightforward, leading to lower costs for large-scale production. At an application rate of 3%, it enables the safe utilization of contaminated soil and adds value to agricultural produce with relatively low investment, demonstrating a favorable input-output ratio. Its effectiveness in immobilizing heavy metals can also reduce subsequent remediation costs, making it attractive from an environmental economics standpoint.

The surface of the tobacco straw biochar is rich in pores, oxygen-containing functional groups, and carbonate ions. These components can reduce the availability of Cd and Pb in the soil through mechanisms such as adsorption, increasing soil pH, and facilitating the formation of insoluble precipitates of heavy metals. Hence, it inhibits the uptake of these metals by pak choi, achieving maximum reductions of 30.76% in Cd content and 38.98% in Pb content in the shoots compared to the control. In addition, tobacco straw biochar enhances SOD activity and decreases MDA content in the leaves of pak choi, improving stress resistance and alleviating the toxic effects of Cd and Pb. In addition, the application of tobacco straw biochar reduces soil BD, increases the content of soil OM, AN, and AK, and enhances both the diversity of soil bacterial communities and the abundance of beneficial bacteria. It also shifts the bacterial community from a stress state dominated by a few tolerant species with highly expressed stress-resistance genes to a recovery state characterized by greater species richness and functional diversity. These improvements create a favorable soil environment for pak choi growth, facilitate the absorption of nutrient elements, and ultimately raise both growth and quality.

### Limitations of the study

The biochar in this study was primarily prepared through pyrolysis in a muffle furnace under a static air atmosphere. While this condition lowers the operational threshold, the presence of oxygen may lead to reduced biochar yield and limited specific surface area, consequently affecting its adsorption and immobilization efficiency for heavy metals. Future research should focus on optimizing material properties by controlling the pyrolysis atmosphere (e.g., using N_2_ protection). As a pot experiment, this study, under controlled conditions, clarified the passivation effect of tobacco straw biochar on soil heavy metals and its growth promotion mechanism for pak choi. However, certain limitations exist. First, due to the inherent structural and compositional characteristics of a single type of biochar, its immobilization effectiveness is selective toward different heavy metals, and its saturation capacity is limited. This makes it challenging to meet the long-term remediation needs of soils with high concentrations or combined contamination. Future work could enhance its remediation efficacy through modification or by combining it with functional microorganisms or clay minerals. Secondly, regarding mechanistic elucidation, further integration with studies such as sequential extraction of heavy metal speciation, analysis of microbial metabolites, and expression of crop nutrient transporter proteins is needed to deepen the mechanistic understanding. Furthermore, pot conditions struggle to simulate complex environmental factors such as field soil heterogeneity, precipitation leaching, and seasonal variations. Subsequent field trials are necessary to validate the uniformity of biochar application, its long-term stability, and its applicability to different soil types, thereby advancing its transition from mechanistic research to practical application.

## Resource availability

### Lead contact

Further information and requests for resources should be directed to and will be fulfilled by the lead contact, Yang Luo (luoyang@gznc.edu.cn).

### Materials availability

This study did not generate new unique reagents.

### Data and code availability

• The raw 16S rRNA gene sequence data for this study have been deposited in the National Center for Biotechnology Information (NCBI) BioProject under accession number PRJNA1399108 and are publicly available immediately without any access restrictions.
• This paper does not report original code.
• Any additional information required to reanalyze the data reported in this paper is available from the lead contact upon request.

## Acknowledgments

This work was supported by the Guizhou Provincial Basic Research Program (Natural Science) General Program (Qiankehejichu MS (2025) 079) and the Scientific Research Fund Project of 10.13039/100018713Guizhou Education University (2026GCC007).

## Author contributions

X.T.: writing – original draft, formal analysis, software, visualization, investigation, data curation, and conceptualization. F.L.: formal analysis and investigation. J.L.: methodology and investigation. Y.L.: writing – review and editing, data curation, conceptualization, and funding acquisition. M.W.: formal analysis and investigation. J.C.: formal analysis and investigation. L.Y.: methodology. X.Y.: software and formal analysis. Z.S.: formal analysis and writing – review and editing.

## Declaration of interests

The authors declare no competing interests.

## STAR★Methods

### Key resources table

**Table:** 

REAGENT or RESOURCE	SOURCE	IDENTIFIER
**Biological samples**		

Soil (Pb/Cd-contaminated lime soil)	Wudang District, Guiyang City, China	Soil sample
Pak choi (Brassica rapa subsp. chinensis cv. Sijiqing)	Guizhou Ruimeng Agricultural Technology Co., Ltd.	Plant material
Tobacco straw	Kaiyang County, Guiyang City	Raw material
Tobacco straw biochar	This study (pyrolyzed at 400 °C for 2 h)	Tobacco straw biochar

**Chemicals, peptides, and recombinant proteins**		

Ethanol	Guizhou Education University	CAS:64-17-5
Acetic acid	Guizhou Education University	CAS:64-19-1
Sodium acetate	Guizhou Education University	CAS:127-09-3
Sodium carbonate	Guizhou Education University	CAS:497-19-8
Hydrochloric acid	Guizhou Education University	CAS:7647-01-0
Glucose	Guizhou Education University	CAS:50-99-9
n-Propanol	Guizhou Education University	CAS:71-23-8
Bovine Serum Albumin	Guizhou Education University	CAS:9048-46-8
Brilliant Blue G-250	Guizhou Education University	CAS:6104-58-1
Activated Carbon	Guizhou Education University	CAS:7440-44-0
Key Commercial Assays	N/A	N/A

**Deposited data**		

Raw 16S rRNA gene sequencing data	NCBI BioProject	PRJNA1399108; https://www.ncbi.nlm.nih.gov/bioproject/PRJNA1399108

**Software and algorithms**		

IBM SPSS Statistics 22	IBM	https://www.ibm.com/products/spss-statistics
Origin 2024	OriginLab	https://www.originlab.com/
PICRUSt2	Published	https://picrust.github.io/picrust/
Mothur	Published	https://mothur.org/

**Table:** Basic physical and chemical properties and contents of Pb and Cd in tested materials

Materials	BD (g/cm^2^)	pH	TOC (g/kg)	AN (mg/kg)	AP (mg/kg)	AK (mg/kg)	TCd (mg/kg)	ACd (mg/kg)	TPb (mg/kg)	APb (mg/kg)
Soil	1.24	7.91	14.74	86.21	37.07	301.51	32.76	13.08	102.34	34.05
Biochar	0.52	9.54	432.15	–	–	–	0.18	–	0.41	–

Note: “-” indicates that the content was not detected.

### Experimental model and study participant details

Soil was collected from Pb/Cd-contaminated lime soil in Wudang District, Guiyang City, China. Pak choi (Brassica rapa subsp. chinensis cv. Sijiqing) was used as the test plant. Tobacco straw was obtained from Kaiyang County, Guiyang City, and pyrolyzed at 400 °C for 2 h to produce biochar. No human or animal subjects were used. Sex-related influences are not applicable to plant–soil systems.

### Method details

#### Test materials

Tested soil: The soil sample was collected from a plot adjacent to a privately owned abandoned coal mine in Wudang District, Guiyang City. This mine had an operational history of approximately 20 years and was shut down in 2007, during which period technical and management measures were inadequate. The long-term mining activities resulted in the open accumulation of waste rock and tailings, leading to the release of heavy metals associated with the raw coal into the surrounding environment. Through prolonged natural weathering and leaching, some elements with higher mobility have undergone transformation or migration. In contrast, Pb and Cd have become enriched due to their stable chemical behavior in the soil, constituting the primary pollutants of focus in this study. The soil type is lime soil, and its basic physicochemical properties and Pb–Cd content are presented in Table 5.

**Table 5:** Bacterial community diversity indices of soils under different treatments

Treatments	Shannon	Chao	Simpson
CK	5.87±0.11b	4353.35±271.49a	0.017±0.002a
B1	6.07±0.04a	4457.51±46.63a	0.014±0.002b
B2	6.09±0.06a	4546.81±50.98a	0.012±0.002b
B3	6.12±0.10a	4227.87±201.04a	0.011±0.002b

Tested tobacco straw biochar: Tobacco straw was collected from a tobacco planting base in Longgang Town, Kaiyang County, Guiyang City. The stalks were dried, ground, and sieved, then placed in a crucible and pyrolyzed in a muffle furnace at 400 °C for 2 h (The temperature and time parameters were primarily determined based on the yield and application effectiveness of the biochar in preliminary experiments). After cooling, the material was passed through a 100-mesh sieve to obtain biochar. Its basic physicochemical properties are listed in Table 1. Meanwhile, the surface morphological characteristics of the tobacco straw biochar were observed using a scanning electron microscope (ZEISS, GeminiSEM 300, Germany). The types of surface compounds were analyzed using an X-ray diffractometer (Rigaku, D/MAX-2600, Japan). The surface functional groups were characterized using a Fourier transform infrared spectrometer (Thermo Fisher Scientific, Nicolet iS20, USA). Thermogravimetric analysis of the biochar was performed using a simultaneous thermal analyzer (HITACHI, STA200, Japan).

Tested pak choi (*Brassica rapa* subsp. *chinensis*): The variety “Si Ji Qing” was purchased from Guizhou Ruimeng Agricultural Technology Co., Ltd. Before use, the seeds were disinfected by soaking in a low-concentration potassium permanganate solution for 30 min.

#### Experimental design

The experiment was conducted from September to November 2024 in the potted plant facility of Guizhou Education University. Following the approach of Ibrahim et al.^51^ and Rehman et al.^52^ four treatments were designed: CK (control), B1 (1% tobacco straw biochar addition), B2 (3% tobacco straw biochar addition), and B3 (5% tobacco straw biochar addition). Each treatment was replicated three times, resulting in a total of 12 pots. The tobacco straw biochar was incorporated into the soil and placed in pots lined with gauze at the bottom. Each pot was filled with 1.8 kg of the soil mixture and conditioned for 15 days at room temperature.

Plump pak choi seeds were selected, and 15 seeds were evenly sown in each pot. When the seedlings developed two true leaves, thinning was performed, leaving two seedlings per pot. During the growth period, the pots were randomly rearranged every three days. Watering was conducted based on weather conditions to maintain soil moisture at approximately 65% of field capacity via the weighing method. The total growth cycle lasted 60 days. At harvest, the shoot parts of the pak choi plants were rinsed with deionized water and dried. A portion of the fresh samples was reserved for the determination of physiological characteristics, while the remaining samples were fixed at 105 °C for 20 min and dried at 70 °C to constant weight for the analysis of heavy metal and nutrient contents. Rhizosphere soil was collected from each pot at the same time (The soil loosely bound to the roots was gently shaken off, and this portion was regarded as the bulk soil. Subsequently, soil that closely adhered to the root surfaces was collected using a sterile brush, and the soil collected in this manner constituted the rhizosphere soil samples). After removing debris, a portion of the fresh soil was used for microbial analysis, while the other portion was air-dried indoors. The air-dried soil was sieved through 2 mm and 0.15 mm meshes for the determination of physicochemical properties and heavy metal indicators.

### Quantification and statistical analysis

#### Growth indicators, MDA and antioxidant enzymes

The leaf width and length of pak choi were measured using a measuring tape, and the fresh weight of the shoot parts was determined using a precision balance with ten-thousandth accuracy. The malondialdehyde (MDA) content in pak choi leaves was analyzed using the thiobarbituric acid colorimetric method,^53^ superoxide dismutase (SOD) activity using the nitroblue tetrazolium colorimetric method,^54^ peroxidase (POD) activity using the guaiacol method,^55^ and catalase (CAT) activity using the ultraviolet absorption method.^56^

#### Quality, nutrient content, and Pb and Cd contents of pak choi

The soluble sugar content in pak choi was measured using the anthrone colorimetric method; vitamin C content was determined by the 2,6-dichlorophenolindophenol titration method; soluble protein content was measured by the Coomassie Brilliant Blue G-250 colorimetric method; and nitrate content was analyzed by ultraviolet spectrophotometry. After digesting the pak choi samples with H_2_SO_4_-H_2_O_2_, the nitrogen (N), phosphorus (P), and potassium (K) contents were determined using the Kjeldahl method, molybdenum-antimony-scandium colorimetry, and flame photometry, respectively. The Cd and Pb contents in pak choi were determined after digestion with HNO_3_-HClO_4_ (3:2, v/v) using an atomic absorption spectrophotometer (novAA350, Analytik Jena, Germany).^57^

#### Soil physicochemical property analysis

Soil bulk density (BD) was determined using the cutting ring method; soil pH was measured with a pH meter (soil-to-water ratio of 2.5:1); soil organic matter (OM) content was analyzed using the potassium dichromate volumetric method with external heating; alkali-hydrolyzable nitrogen (AN) was determined by the alkali diffusion method^58^; available phosphorus (AP) was extracted with 0.5 mol/L NaHCO_3_ and measured using molybdenum-antimony anti-colorimetry; available potassium (AK) was extracted with NH_4_OAc and determined by flame photometry; available Cd (ACd) and Pb (APb) contents in soil were extracted with 0.1 mol/L HCl and measured by atomic absorption spectroscopy^57^; and the soil bacterial community structure was analyzed by Sangon Biotech (Shanghai) Co., Ltd.^59^

#### Statistics analysis

All data obtained from the experiment were processed using Microsoft Excel 2021 and are presented as mean ± standard deviation. Significance analysis among treatments was conducted using the Least Significant Difference (LSD) method in IBM SPSS Statistics 22.0. Graphs were prepared using Origin 2024. The ACE, Shannon, and Simpson indices of soil bacteria were calculated using Mothur. The metabolic functions of the bacterial communities were predicted using the PICRUSt 2 software.
